# The diversity, development and evolution of polyclad flatworm larvae

**DOI:** 10.1186/2041-9139-5-9

**Published:** 2014-03-06

**Authors:** Kate A Rawlinson

**Affiliations:** 1 Biology Department, Dalhousie University, 1355 Oxford Street, Halifax NS B3H 4R2, Canada

**Keywords:** Polyclads, Platyhelminthes, Larvae, Meroplankton, Lobes, Ciliary band, Sucker

## Abstract

Polyclad flatworms offer an excellent system with which to explore the evolution of larval structures and the ecological and developmental mechanisms driving flatworm and marine invertebrate life history evolution. Although the most common mode of development in polyclads might be direct development (where the embryo develops directly into a form resembling the young adult), there are many species that develop indirectly, through a planktonic phase with transient larval features, before settling to the sea floor. In this review, I introduce polyclad life history strategies, larval diversity and larval anatomical features (presenting previously unpublished micrographs of a diversity of polyclad larvae). I summarize what is known about polyclad larval development during the planktonic phase and the transition to the benthic juvenile. Finally, I discuss evolutionary and developmental scenarios on the origin of polyclad larval characters.

The most prominent characters that are found exclusively in the larval stages are lobes that protrude from the body and a ciliary band, or ciliary tufts, at the peripheral margins of the lobes. Larvae with 4–8 and 10 lobes have been described, with most indirect developing species hatching with 8 lobes. A ventral sucker develops in late stage larvae, and I put forward the hypothesis that this is an organ for larval settlement for species belonging to the Cotylea. Historically, the biphasic life cycle of polyclads was thought to be a shared primitive feature of marine invertebrates, with similarities in larval features among phyla resulting from evolutionary conservation. However, our current understanding of animal phylogeny suggests that indirect development in polyclads has evolved independently of similar life cycles found in parasitic flatworms and some other spiralian taxa, and that morphological similarities between the larvae of polyclads and other spiralians are likely a result of convergent evolution.

## Review

### Introduction

**Figure 1:**
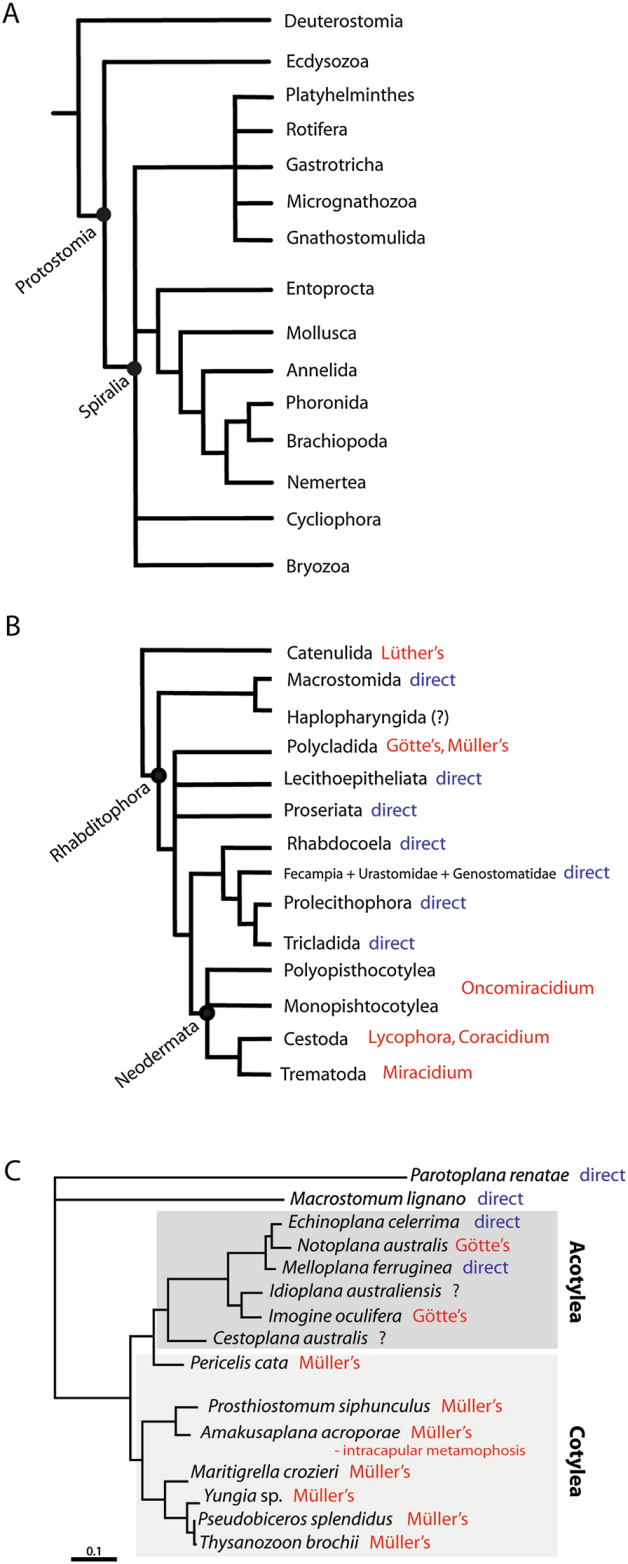
**Phylogenetic relationships of the Spiralia and the Platyhelminthes. (A)** Spiralians [9]. **(B)** Platyhelminthes [10]. **(C)** A skeleton phylogeny of polyclads from a Bayesian analysis of 28S rDNA sequence data [5]. Mode of development of platyhelminth groups and polyclads indicated in red (free-swimming ciliated larvae) and blue (direct development). Question mark (?) indicates mode of development is unknown.

The Platyhelminthes (flatworms) are an extremely diverse spiralian clade (over 100,000 known species [1]) (Figure 1A, B) that includes the catenulids and rhabditophorans but excludes the acoels and nemertodermatids [2]. There are two groups of flatworms that disperse via a ciliated, swimming larval stage: the parasitic neodermatans, and the free-living polyclads. Neodermatans have evolved very elaborate life cycles, often with multiple, morphologically distinct larval stages. The first larval stages of the various neodermatan life cycles (for example, the oncomiracidium of the Polyopisthocotylea and Monopisthocotylea, the miracidium and cotylocidium of the digenean and aspidogastreans trematodes respectively, and the lycophore and coracidium of the cestodes [3,4]) swim using plates or bands of ciliated epidermal cells. Polyclads are marine predatory flatworms found generally on the seafloor. They feed on a wide variety of marine invertebrates (see references in [5]) and a few have symbiotic relationships with other animals [6,7]. Like many benthic marine invertebrates, polyclads exhibit a diversity of life history strategies spanning direct development (the embryo develops directly into a form resembling the young adult), intermediate development (the embryo develops through an encapsulated larval stage and hatches in the form of a young adult) and indirect development (the embryo develops indirectly into the young adult form through a planktonic larval stage). Polyclad larval stages (known as Götte’s and Müller’s larvae) swim using ciliary bands, or ciliary tufts, placed on protrusions of the body wall. All other flatworms have direct development, with the exception of one freshwater species of catenulid, which swims at hatching using several anterior rings of elongated cilia but that in all other respects resembles the adult; this swimming stage is known as a Luther’s larva [8].

Jägersten [11] considered the biphasic life cycle of polyclads to be the ancestral condition for Platyhelminthes. He also considered polyclad larvae to be primary larvae (a free-swimming larval stage that was descended directly from that of the last common ancestor of the metazoans) and neodermatan larvae as secondary larvae (a larval phase evolved secondarily from a direct-developing ancestor). In other words, polyclad and neodermatan larvae were thought to have evolved independently, with polyclad larval characters being primitive features shared between phyla with primary larvae (for example, cyphonautes (Bryozoa), trochophora (Annelida, Mollusca), actinotrocha (Phoronida), tornaria (Hemichordata), auricularia (Echinodermata)), and neodermatan larval characters having evolved *de novo*. Over the last four decades, our knowledge of metazoan phylogeny and larval morphology has increased significantly, and we can now revisit these hypotheses in light of new data. This review examines polyclad larval diversity, development and evolution by summarizing classical literature, synthesizing recent work and presenting new data. This review also establishes a foundation for future studies of metazoan larval character evolution.

### Polyclad life history strategies and larval diversity

Polyclads are an under-studied order of flatworms. Most polyclad species are rare [12], and the bulk of published literature consists of species descriptions. There are approximately 800 described species of polyclads [13], and the presence or absence of a sucker on the ventral surface of the adult has been used historically to distinguish between polyclad suborders [14]: acotyleans (approximately 450 species) generally lack a sucker, whereas cotyleans (approximately 350 species) possess a sucker at varying positions along the ventral midline posterior to the female gonopore. Over the past 130 years, a handful of studies on polyclad reproduction and development have described the mode of development in approximately 63 species; 39 acotyleans and 24 cotyleans (Table 1; and Table 1 in [15]). All polyclads are hermaphrodites and have internal fertilization. Polyclad embryos are released onto the seafloor in protective capsules, and hatch with a ciliated epidermis. Patterns of ciliation and body shape differ depending on whether a species hatches as a larva (indirect development) or as a juvenile (direct development). At hatching, indirect-developing species have complex body shapes with projections (lobes, lappets or arms) from their bodies and additional cilia collected into distinct bands (or tufts), at the edges of these protrusions for locomotion and feeding. Conversely, direct developers hatch with a simple, dorso-ventrally flattened, prolate spheroid body shape and no accessory cilia. Species with intermediate development develop larval features but undergo metamorphosis before hatching. Arguably, direct development may be the more common mode of development within polyclads, given that this strategy is more prevalent among acotyleans [15], which in turn, are more species-rich than cotyleans. That said, a biphasic life cycle is widespread and larval stages are morphologically diverse.

**Table 1:** Polyclad species with indirect development from published literature

**Suborder, family**	**Species**	**Number of larval lobes**	**Larval type**	**Length at hatching, μm**	**Reference**
**Acotylea:**					
**Stylochidae**	*Imogine aomori*	4	Götte’s	95 to 105	[6]
	*Imogine ijimai*	4	Götte’s		[16]
	*Imogine lateotentare*	4	Götte’s		[17]
	*Imogine mcgrathi*	4	Götte’s	150	[18]
	*Imogine mediterraneus*	4	Götte’s	90	[19,20]
	*Imogine uniporus*	4	Götte’s	85 to 95	[6]
	*Stylochus ellipticus*	4	Götte’s	90	[14,21] this review
	*Stylochus flevensis*	4	Götte’s		[22]
	*Stylochus pilidium*	4	Götte’s		[14]
	*Stylochus pygmaeus*	4	Götte’s		[23]
	*Stylochus tauricus*	4 to 5	Götte’s	90 to 100	[24]
**Notoplanidae**	*Notoplana australis*	4	Götte’s	90	[25]
**Leptoplanidae**	*Hoploplana inquilina*	6	Müller’s	90	[26]
**Planoceridae**	*Planocera multitentaculata*	8	Müller’s	170 to 185	[6]
	*Planocera reticulata*	7	Müller’s: intracapsular & free-swimming (known as Kato’s larva)	310 to 330	[6,27]
**Cotylea:**					
**Pseudocerotidae**	*Pseudoceros canadensis*	6	Müller’s	150	[28,29]
		8			[30]
	*Thysanozoon brochii*	8	Müller’s	180	[20,31]
	*Yungia aurantiaca*	8	Müller’s		[14]
	*Pseudoceros maximus*				[14]
	*Pseudoceros vittatus*				[14]
	*Pseudoceros bicolor*	8	Müller’s	200	*Pers obs*
	*Phrikoceros mopsus*	8	Müller’s	160	*Pers obs*
**Euryleptidae**	*Maritigrella croizeri*	8	Müller’s	250	[32]
	*Oligocladus auritus*				[33]
	*Cycloporus papillosus*				[14]
	*Cycloporus variegatus*		Müller’s	150	*Pers obs*
	*Eurylepta cornuta*				[14]
	*Eurylepta lobianchii*				[14]
	*Eurylepta leoparda*	8	Müller’s		[34]
	*Eurylepta lobianchii*				[14]
	*Stylostomum ellipse*				[14]
	*Stylostomum sanjuania*	8	Müller’s	120	[29]
	*Prosthecereus giesbrechtii*	8	Müller’s		[35]
**Prosthiostomidae**	*Prosthiostomum siphunculus*	8	Müller’s	180	[14,20]
	*Prosthiostomum auratum*	8			
	*Prosthiostomum montiporae*	8	Müller’s		[36]
	*Amakusaplana acroporae*	8	Müller’s: intracapsular	250 to 300	[5]
**Unknown**	?	10		300	This review
**Unknown**	?	10		5000	[11]

*Pers obs*, personal observation.

Culturing polyclad larvae to metamorphosis is difficult and rarely achieved, so much of our understanding of this life history stage has come from plankton tows. For example, development over the larval stage and metamorphosis has been illustrated based on specimens collected from the plankton [14,37], and a higher diversity of larval forms have been captured in plankton nets [11,38,39] than have been described from laboratory cultures. The water column is a relatively under-sampled environment for polyclads, which generally undergo embryogenesis and spend their adult life on the sea floor. Consequently, sampling coastal waters (which are rich in meroplankton - the larval stages of organisms that will become nektonic or benthic as adults) may lead to the discovery of new larval types, an increased understanding of the developmental processes that take place over the planktonic stage, and new hypotheses on the evolution of biphasic life cycles.

**Figure 2:**
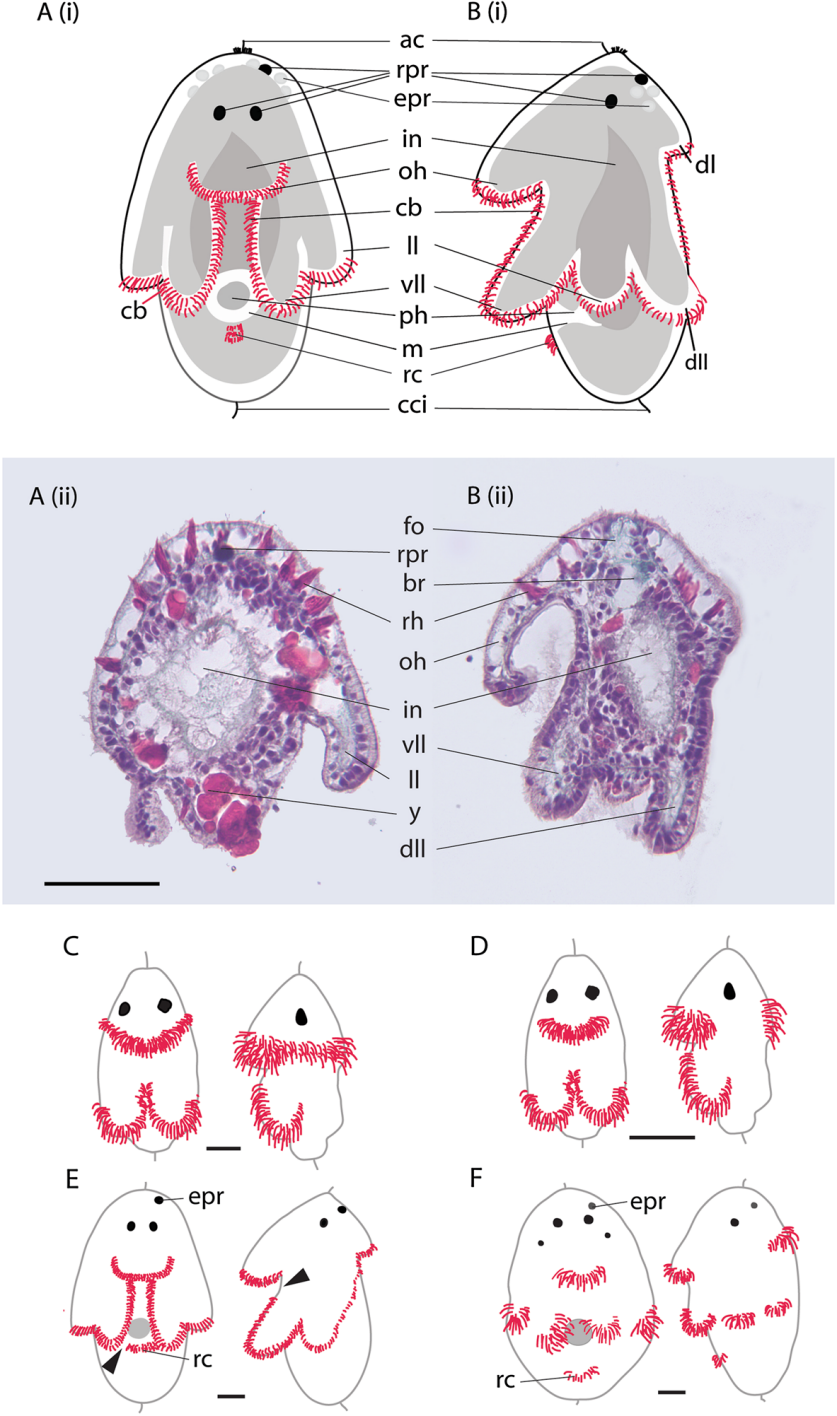
**Schematic diagrams and histological sections showing the gross anatomy of an eight-lobed larva and variation in ciliary band and ciliary tuft configurations (red) among polyclad larvae. (A, B)***Maritigrella crozieri* (2 days post hatching)*.***(A) (i)** Ventral view, **(ii)** frontal section; **(B) (i)** left lateral view, **(ii)** sagittal section. Masson’s trichrome stain, scale = 100 μm. **(C-F)** Ventral and left lateral views of *Imogine mcgrathi***(C)**, *Stylochus ellipticus***(D)**, *Pseudoceros canadensis***(E)**, *Amakusaplana acroporae***(F)** larval stages, scale = 25 μm. **(C)***I. mcgrathi* Götte’s larva with a continuous ciliary band anterior to the mouth, and separate ciliary bands on the ventro-lateral lobes that continue medially towards the mouth [45]. **(D)***S. ellipticus* Götte’s larva with discrete ciliary tufts on the oral hood, dorsal lobe and ventro-lateral lobes (personal observation). **(E)***P. canadensis* Müller’s larva with a single but discontinuous band, breaks in ciliary band indicated with arrowheads [28]. **(F)***Amakusaplana acroporae* intracapsular Müller’s larva with discrete ciliary tufts on eight short lobes [5]. ac, apical cilia; br, brain; cci, caudal cilia; epr, putative extraocular photoreceptor cells; dl, dorsal lobe; dll, dorsolateral lobe; fo, frontal organ; in, intestine; ll, lateral lobe; m, mouth; oh, oral hood; ph, pharynx; rc, putative rejectory cells; rh, rhabdites; rpr, rhabdomeric photoreceptor cells; vll, ventrolateral lobes; y, yolk.

**Figure 3:**
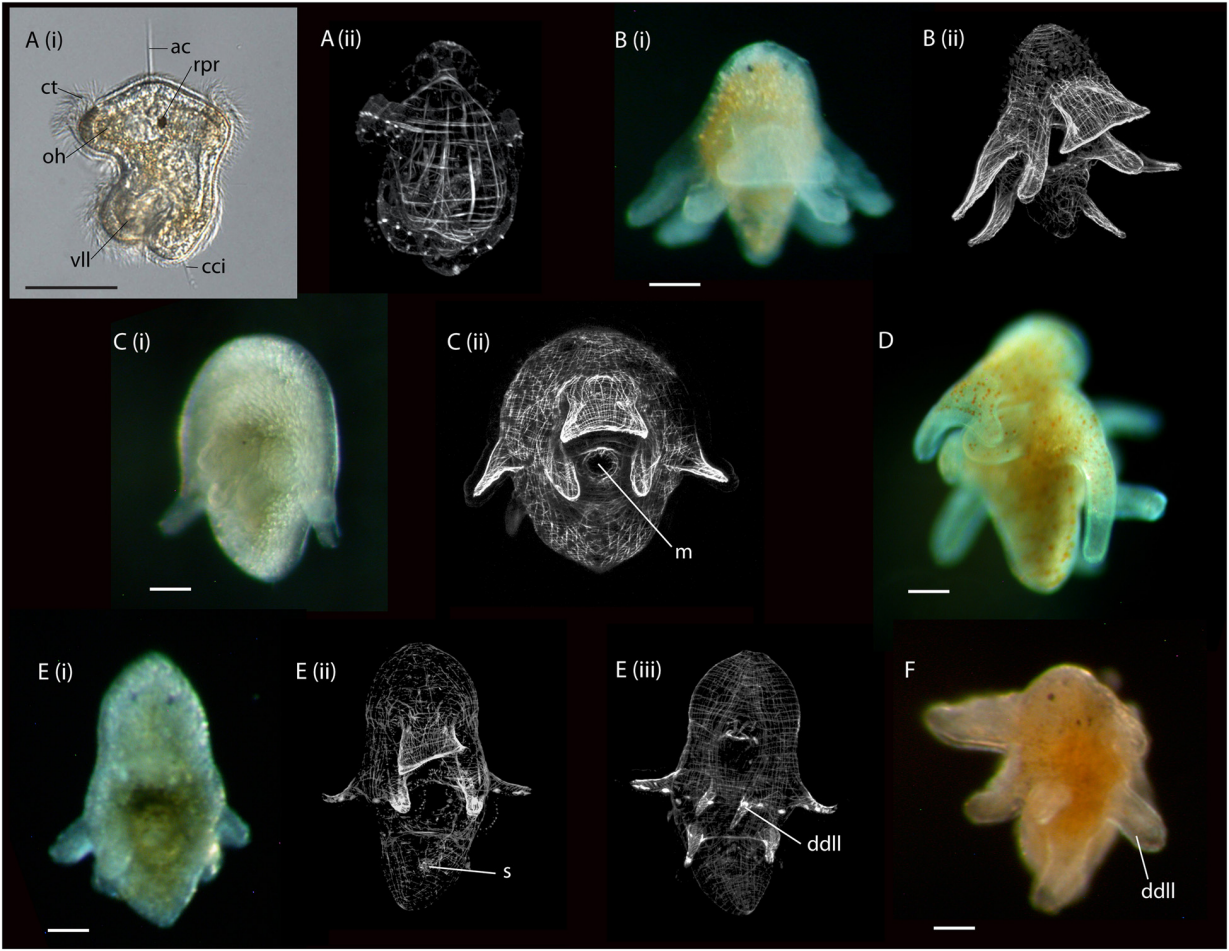
**Photomicrographs of live polyclad larvae and confocal laser scanning micrographs of their musculature stained with phalloidin. (A)** Götte’s larva of *Stylochus ellipticus*, left lateral view. **(B-F)** Müller’s larvae collected from plankton offshore of Fort Pierce, Florida. **(B-D)**: 8-lobed larvae; **(E, F)** 10-lobed larvae. In **(E)** the late larval stage of this worm is recognizable by elongation in the anterior-posterior axis, dorso-ventral flattening and the development of a sucker on ventral surface. The lateral lobes are relatively short and are slowly being incorporated back into the body. *ac*, apical cilia; *cci*, caudal cilia; *ct*, ciliary tufts; *ddll*, paired dorsal lobes; *m*, mouth; *oh*, oral hood; *rpr*, rhabdomeric photoreceptor, *s*, sucker; *vll*, ventrolateral lobes. Scale = 50 μm.

Classically, polyclad larval stages have been divided into two groups based on the number of lobes: the Götte’s larva having four lobes [6,22,40-42] and the Müller’s larva with eight lobes [14,43,44]. However, as larvae with different numbers of lobes were subsequently discovered they were assigned to one group or the other; consequently there is a Götte’s larva with five lobes [24], and Müller’s larvae with six, seven and ten lobes [6,26,27,38,39] (Table 1). These larval names may not have any phylogenetic significance, and with further investigation into the anatomical structures of these larvae and a greater understanding of larval character diversity and polyclad phylogeny, these terms will probably be redefined or become obsolete. However, for the sake of this review, I will continue to use the terms Götte’s (four to five lobes) and Müller’s (six to eight and ten lobes) larva in the classical sense. All polyclad larvae have an oral hood, a dorsal lobe (except *Planocera reticulata*[6]) and a variable number of paired lateral lobes (Figure 2). Götte’s larvae are small (size range 85 to 150 μm in length, Table 1) and simple in body shape, with one pair of short lateral lobes that sit either side of the mouth (Figure 3A). Müller’s larvae are larger (size range 90 to 5,000 μm in length, Table 1), with two to four pairs of long lateral lobes contributing to a more complex body shape (Figure 3B-F).

Lang [14] proposed that the Götte’s larva was an earlier developmental stage of the Müller’s larva, with two more pairs of lateral lobes developing during the larval phase. However, it is now widely accepted that the Götte’s larva is a distinct larval form from the Müller’s larva, as many species that hatch with four lobes never develop any further lobes [6,25] (with the exception of *Stylochus tauricus* which was described to develop a fifth lobe 7 days post hatching [24], although it is not clear from their illustrations where this lobe is positioned). There is evidence that in some Müller’s larvae, further lobes may develop over the larval period. For example, at hatching, the cotylean *Pseudoceros canadensis* has six lobes [28,29] but a pair of dorso-lateral lobes develops later [30]. Furthermore, there are no observations of species hatching with ten lobes, which led Dawydoff [38] to speculate that plankton caught with ten lobes hatch with eight, and gain additional lobes during the larval period.

**Figure 4:**
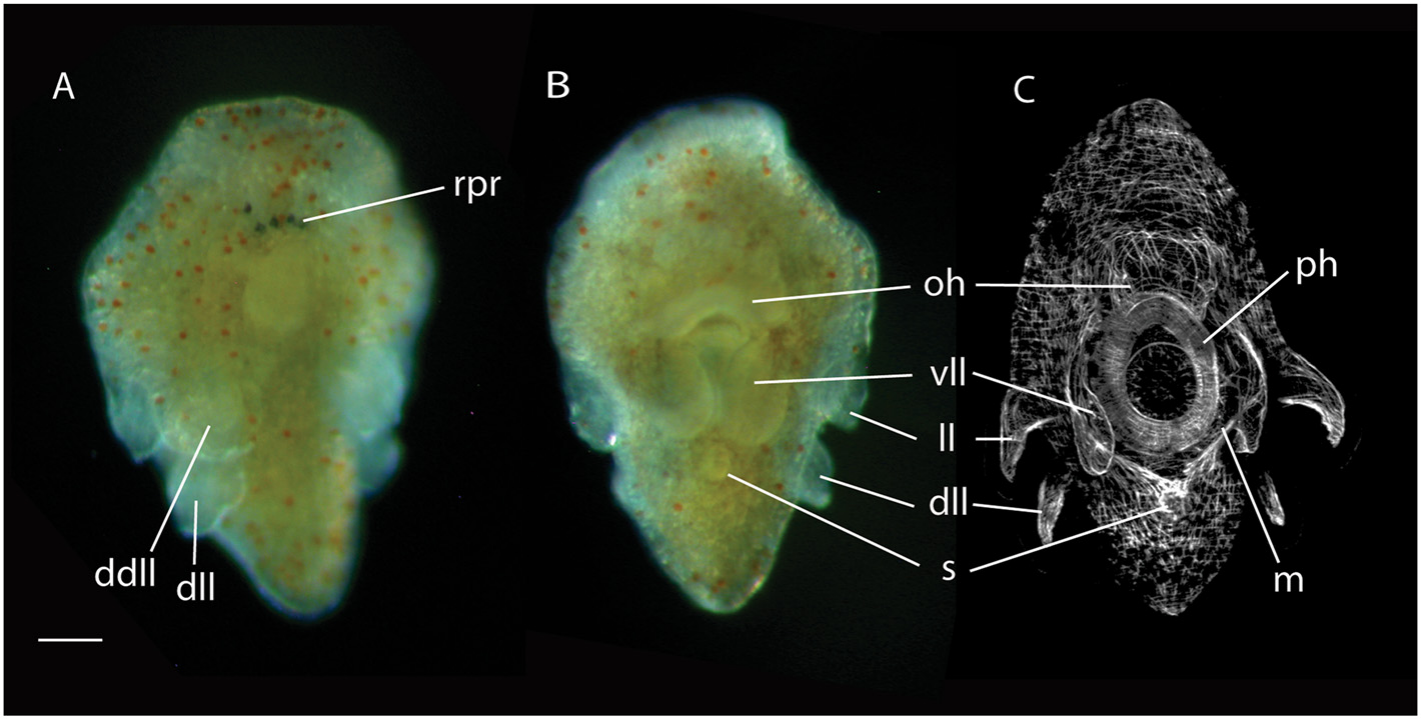
**Photomicrographs of a late stage 10-lobed larva collected from the plankton. (A)** Live animal - dorsal view showing five rhabdomeric photoreceptors and the paired dorsal and dorsolateral lobes. **(B)** Live animal - ventral view reveals a well-developed sucker posterior to the mouth. **(C)** Confocal laser scanning micrograph of the same larva stained with phalloidin showing the musculature of the sucker, mouth and developing pharynx. Scale = 50 μm. *dll*, dorsolateral lobe; *ddll*, paired dorsal lobes; *ll*, lateral lobes; *m*, mouth; *oh*, oral hood; *ph*, pharynx; *rpr,* rhabdomeric photoreceptors; *s*, sucker; *vll*, ventrolateral lobes.

All cotylean species develop via an eight-lobed larva, whereas, most indirect-developing acotyleans develop via a four-lobed larva (though larvae with five, six and seven lobes also occur; Table 1). The taxonomic affinity of the 10-lobed larvae has not been investigated [11,38], but the presence of a sucker posterior to the mouth (Figure 3Eii and 4B, C) would suggest that they are cotyleans (see Section 3). These larvae have only ever been collected from the plankton, and Figure 3E-F and Figure 4 represent the first photographs of this larval form. It is also worth noting that 10-lobed larvae were more common than 8-lobed larvae in plankton tows in the Gulf Stream off Florida. Intermediate development has been recorded in two species (*Planocera reticulata*[6] and *Amakusaplana acroporae*[5]), whereby the embryos develop larval characters but undergo intracapsular metamorphosis hatching as juveniles. In *Planocera reticulata*, however, there appears to be variation in the time of hatching, with some individuals emerging as a lobed larva [27].

### Polyclad larval characters: morphology, development and function

The larval stages of benthic marine invertebrates have classically been regarded as morphologically, ecologically and physiologically distinct from the juvenile and adult stages. However, it is now clear that in some spiralian taxa, many features of the larval body plan persist into the adult body plan, with the larval period defined by the presence of a collection of transient “larval characters” [46-49]. With respect to polyclad flatworms, we are in the early stages of determining which characters are specific to the larval stage. The most obvious larva-specific features are the lobes and ciliary bands or tufts. Other characters, such as the frontal (or apical) organ, are also reported to be lost during metamorphosis [6,37]. More detailed studies on the metamorphosis of indirect-developing species, and comparative analyses of indirect and direct-developing taxa, are needed to distinguish between characters that are unique to the larval stage, and those that persist through the larval stage and into the juvenile/adult.

Larval lobes are temporary protrusions [14] made up of epidermal cells (including prototrochal cells, ciliated epidermal cells, monociliated sensory cells and neurons [37]), body wall muscle [50,51] and connective tissue/extracellular matrix [50] (Figure 2B). Unlike the rest of the animal the lobes lack epidermal rhabdites and yolk [50] (Figure 2B). Outgrowth of the lobes occurs relatively late in embryogenesis (during the last 24 hours prior to hatching in *Maritigrella crozieri*, [52]). The long lobes found in larvae collected from the plankton would suggest that lobe growth continues in the water column (Figure 3B, D, F). The cellular mechanisms that drive the outgrowth of the lobes are unknown, as is the fate of lobe tissue during resorption/settlement. The body wall muscle (and other tissue) of the lobes may be incorporated into the body of the juvenile and adult.

At the peripheral margins of the lobes, cells with longer cilia form continuous, pre-oral ciliary bands (often called prototroch) (such as *Maritigrella crozieri,*[52,53]), discontinuous bands (*Pseudoceros canadensis*[28]), or numerous distinct bands or tufts (*Imogine mcgrathi*[45]; *Amakusaplana acroporae*[5]) (Figure 2C-F). Further analysis is needed to investigate the variation in the configuration of the ciliary bands among species, and to assess its functional significance. In *M. crozieri*, the ciliary band is formed by hundreds of prototrochal cells, positioned in rows [53] and innervated by a dense sub-cilial plexus of serotonin and FMRFamide immunoreactive cells [50]. The ciliary band forms shortly after gastrulation as a ring around the developing stomodaeum at the posterior end of the embryo (in *M. crozieri*; [52]). As the stomodaeum migrates ventrally, the ciliary band becomes convoluted and migrates to the position of the future larval lobes. In *P. canadensis* cells with longer cilia are also described from the sub-oral plate. They are continuous with the ciliary band that follows the lobes and Lacalli [54] calls these rejectory cells and assumes a function in food rejection (Figure 2E). It is possible that these cells are found in *M. crozieri* (see Figure seven D in [53]) and *A.acroporae* (Figure six D in [5]) larvae as well (Figure 2A, F).

Polyclad larvae swim continuously [28], and metachronal waves propagate around the ciliary band providing the main propulsive force [55]. The ciliated bands are also used for feeding; with cilia on the lobes beating in the direction of the larval body, cilia on the ventral surface beating towards the mouth and specialized club-shaped cilia hypothesized to function as a valve in a ring at the stomodaeum-gut junction [37].

Although there are no direct observations of Müller’s and Götte’s larvae feeding, phytoplankton was observed in the gut lumen of eight-lobed Müller’s larvae [30,56], with greater survival times in fed compared to unfed larvae [56]. Götte’s larvae, on the other hand, are considered lecithotrophic [25,37], or facultative planktotrophs (it has been reported that *Imogine mcgrathi* feeds passively on microplankton [45]). Comparisons of body shape and placement of cilia on marine larval bodies reveal that there is a tendency for feeding larvae to have complex body shapes, and for nonfeeding larvae to have simple body shapes [57]. These correlations of body shape with trophic status seem to apply for polyclad larvae: Müller’s larvae, with complex and large body shapes, are probably active planktotrophs, whereas the small, simple Götte’s larvae are facultative feeders or lecithotrophs.

At the anterior pole of polyclad larvae is a tuft of cilia and associated gland cells. These have been named a frontal organ [6,37], apical organ [28] or apical plate [45,52], and a glandulo-sensory function has been suggested [37]. We do not have a very complete picture of this organ’s anatomy and function, as different studies have used different methods and different species, so comparison across polyclad taxa is difficult. I shall call this organ a frontal organ to avoid suggesting that it is homologous to the apical organ found in other spiralian larval stages, as we do not have sufficient data yet to make these comparisons. Histologically, the frontal organ looks like a columnar mass of connective tissue between the brain and the apical cilia [6] (Figure 2Bii). Ultrastructural studies [28,37,58] show that the frontal organ consists of a cluster of monociliated or multicilated sensory cells (in Müller’s and Götte’s larvae respectively [37]), surrounded by a circle of gland-cell necks supported peripherally by microtubules. The gland-cell necks pass through the basement membrane extending to secretory-cell bodies around the brain [28,37]. The bases of the monociliated sensory cells in *Pseudoceros canadensis* are located on top of the brain, but no direct connection to the brain has been observed. Lacalli [28] therefore determined that these cells were not nerve cells. Immunohistochemical and immunofluorescent studies [45,52] show immunoreactivity in the form of an apical plate. Antibodies against the neurotransmittors serotonin (5HT) and FMRFamide are expressed in a ring around the apical cilia in *M. crozieri*[52] and these expression patterns may correspond to the monociliated sensory cells of *P. canadensis*[58]. There appears to be little 5HT or FMRFamide immunoreactivity in the rest of the organ [52]. These preliminary findings may suggest that the frontal organ may be more glandular than sensory, but that it is surrounded by neural activity in the brain and apical plate.

Kato [6] suggested that the frontal organ is used to break the eggshell at hatching and noted that it degenerates 1 to 2 days post hatching in *Planocera reticulata* (which has already undergone metamorphosis inside the egg capsule). If the frontal organ does have a role in breaking the eggshell, then perhaps it is also found in direct-developing hatchlings. Apical cilia have been identified in direct-developing hatchlings [15] and comparative studies between direct- and indirect-developing species would determine whether the frontal organ is a character unique to indirect developers. Ruppert [37] suggests that the frontal organ may play a role in larval settlement, similar to the organ of larval attachment in larvae of the ectoproct *Loxosomella*. There are many fundamental questions on the morphology, function and evolution of the frontal organ in polyclads that need to be answered. Ideally, in one species, we would use scanning and transmission electron microscopy, immunostaining and gene expression analysis to understand the relationship between the apical cilia, the gland cells and their innervation. We would also carry out experiments to test whether the frontal organ functions as a specialized sensory-neurosecretory structure that detects environmental cues and transduces them into behavioral responses (for example, settlement).

At hatching, ciliary-band beating and activity of longitudinal, circular and diagonal muscles to steer the larvae are coordinated by sensory and motor nerves centralized through the brain [52]. Light-sensing organs in the form of pigmented rhabdomeric photoreceptors and putative non-pigmented extra-ocular photoreceptors [28,45,52] (Figure 2) detect light information, allowing the larva to assess its depth, detect shadows of predators and swim accordingly. These light-sensing organs are, however, not specific to the larval phase and become more numerous and modified in the juvenile and adult stages ([59] and Rawlinson (unpublished data)). A study on polyclad larval photobehavior demonstrated that *M. crozieri* larvae are relatively insensitive to light (compared to other invertebrate larvae) but that their visual sensitivity increases with age [60]. They display an ontogenetic pattern in which young larvae are positively phototactic at high light-intensities and negative at low (a pattern typical of a predator avoidance shadow-response, which describes an animal’s defensive response to a sudden decrease in illumination), while older larvae are only positively phototactic (a behavior suggested to be beneficial for use of near surface tidal currents for recruitment to shallow adult habitats) [60].

### Development in the water column and transition to the benthic juvenile stage

Planktonic larvae commonly pass through an extended period of development before they become competent to settle and metamorphose [61]. Much of the growth of feeding larvae occurs during this precompetent period [61-63]. Is feeding necessary for polyclad larvae to grow and achieve metamorphic competence? In the absence of food, the four-lobed larvae of *Notoplana australis* and *Stylochus uniporus* settled and metamorphosed after a short planktonic phase (two weeks and 4 to 5 days respectively [6,25]). For Müller’s larvae, yolk is still present at hatching (Figure 2), so perhaps there is a period of facultative planktotrophy (and possibly obligatory planktotrophy, depending on the duration of the larval stage and extent of yolk reserves in different species) [14,37]. Müller’s larva of some species may spend prolonged periods in the plankton [38]. The larval period for *M. crozieri* is estimated to be at least 3.5 weeks [60], and at least 40 days for *P. canadensis* and *Stylostomum sanjuania*[29]. For species with intermediate development, intra-capsular larvae are lecithotrophic [5,6] and adelphophagy has been observed [6].

The transition from a slightly laterally flattened planktonic larva to the dorso-ventally flattened benthic adult involves changes in the mode of feeding and locomotion, from ciliary to muscular in both cases. This transition in polyclads is gradual [14,37], and involves the development of the gut diverticula, a plicate pharynx, and diagonal body wall, dorso-ventral, parenchymal and sucker muscles [37,52]. In some marine invertebrate larvae, metamorphic competence is reached when all requisite juvenile characters are present in the larva, and thus metamorphosis is essentially the loss of larva-specific structures and physiological processes [64]. We know so little about development in the larval and early juvenile stages that we have no understanding of larval competence in polyclads. However, evidence of competence might be the presence of a developed sucker on the ventral surface in some species (see below) and changes in phototactic behavior. Some lecithotrophic species may be competent at hatching, as is typical of most lecithotrophic Porifera, Cnidaria, Bryozoa with coronate larvae, colonial Ascidiacea and many polychaetes [64].

A caudal adhesive organ (sucker) develops prior to metamorphosis in cotylean polyclads (*Thysanozoon* and *Yungia* species; [14,37]), and this was found commonly in eight- and ten-lobed larvae collected from the plankton (Figures 3Eii and 4). No hatchling or early larval stages have been reported to have a sucker, so this organ probably develops later in the planktonic phase. While its function in adult cotylean species is suggested to be a holdfast against the action of waves and currents [65], its role in larval stages has not been investigated. I put forward the hypothesis that the cotylean sucker is a larval character used for attachment during settlement and metamorphosis, and that it has been retained in the adult as a holdfast. Furthermore, I propose that cotyleans without suckers have lost their suckers because they no longer have a distinct pelagic larval stage. Evidence to support this hypothesis comes from *A. acroporae*, a sucker-less cotylean that develops larval lobes and ciliary tufts inside the egg capsule but undergoes metamorphosis before hatching [5]. *A. acroporae* no longer has a free-swimming larval stage and no longer develops a sucker for attachment during settlement and metamorphosis. In order to test this hypothesis, the life history strategies of the seven other cotylean species known to lack ventral suckers [66] would have to be investigated, and I would predict that they have lost a larval stage either developing directly or via an encapsulated larva. Do the larval stages of acotylean species possess a sucker to attach to the substrate at settlement? This has not been investigated or noted, and is unlikely. Although there are a few acotyleans that have adhesive organs (genital suckers: *Leptoplana tremellaris, Itannia ornata*; adhesive discs: cestoplanids), all acotylean species known to have a larval stage do not have suckers as adults.

All that we know about polyclad settlement behavior comes from observations of larvae in culture. They become demersal, whirl in circles on the substrate, come to a halt and contract their bodies into various shapes [14,30]. Some Götte’s larvae become smaller and partially reabsorb their lobes [37], though this behavior is considered pathological [22,39], based on the presence of necrotic cells [37]. There are only three reports of larvae settling and undergoing metamorphosis in culture - *N. australis*[25], *S. uniporus*[6] and *M. crozieri*[60] - but these were casual observations, and the morphology of the settling larvae was not studied in detail. My observations of late-stage larvae collected from the plankton (for example, Figures 3E and 4 in their elongated, dorso-ventrally flattened form with reduced lobes) reveal that their morphology is very plastic. These larvae can crawl on the bottom of a Petri dish, but can also contract, becoming spherical and lobed again before swimming off readily. This ability to alternate between swimming and crawling could allow the larva to sample different substrates before losing the lobes and ciliary band at metamorphosis.

The only data available on morphological changes during metamorphosis come from larvae collected from the plankton [14,37], and from *P. reticulata*, which undergoes intra-capsular metamorphosis [6]. In *P. reticulata,* the lobes, ciliary band and the apical and caudal sensory cilia are lost over 10 days (inside the egg capsule), and the frontal organ is lost shortly after (post hatching) [6]. Ruppert [37] observed the gradual dwindling, reduction and reabsorption of lobes into the body wall and the incorporation of the prototrochal cells into the juvenile epidermis. Over this time many of the organs that persist in the juvenile and adult develop further, for example, intestinal branches develop, eyes increase in number and the simple larval pharynx becomes a plicate pharynx [6,11,37]. The body becomes dorso-ventrally flattened, elongated and thin with prominent growth in the anterior half [6]. Eyes present in the larvae persist into the juvenile and adult, although with modifications. The epidermal eye conceivably sinks into the parenchyma and changes into a cerebral eye with the addition of two ganglionar cells [59]. The cilia of the epithelial pigmented cell persist in the larval cerebral eye, while they disappear in the cerebral eye of the adult [59]. Due to the difficulty of culturing polyclad larvae to settlement and metamorphosis [29,56], and until larval husbandry techniques improve, species with intermediate development provide a rare opportunity to study polyclad metamorphosis. *A. acroporae* (the *Acropora*-eating polyclad) is of particular interest, due to its accessibility (a common pest in reef tanks) and its more typical eight-lobed larval morphology [5].

### Phylogenetic and developmental scenarios on the evolutionary origins of polyclad larval characters

Polyclad larval features bear some similarity to larval characters of parasitic (neodermatan) flatworms and more distantly related spiralians. The first larval stages of neodermatans have, for example, ciliated epidermal cells placed in zones (oncomiracidium larvae, [3]) or on ridges (*Schistosoma mansoni* miracidium, [67]) for swimming to locate a host. They have a sucker (haptor) for attachment during metamorphosis [68], pigmented and putative unpigmented photoreceptors, and ciliary sensila that are involved in phototaxis, geotaxis and rheotaxis [3]. Ciliary bands for locomotion and feeding and sensory apparatus for orientation in the water column are also found in many spiralian and metazoan larvae. Morphological similarities in these larval features led to the suggestion that they are evolutionarily conserved and that the biphasic life cycle is a shared primitive feature of marine invertebrates [11,69,70]. Cell lineage data were seen to support this idea, with polyclad and other spiralian larval ciliary bands considered homologous due to similarities in blastomere contribution [71]. However, recent hypotheses of animal phylogeny do not lend support to this evolutionary scenario, and new data from cell lineage studies are revealing variation in the fate maps of spiralian blastulae [48,72] that makes comparing cell fates between polyclad and other spiralians difficult.

Our current understanding of animal and flatworm phylogeny (Figure 1A, B [9,10]) shows that the sister taxa of polyclads are all direct-developing. This suggests that indirect development in polyclads has evolved independently of similar life cycles found in parasitic flatworms and some other spiralian taxa. It follows then that morphological similarities in larval characters between polyclads, neodermatans and other spiralians are likely a result of parallel or convergent evolution. Morphological and molecular comparisons of these larval features may shed light on whether these features represent the redeployment of conserved cell types, or the convergent invention of similar structures. The argument for homology of anatomical structures between polyclads and other spiralians based on similar blastomere origins is problematic because it is difficult to identify the embryonic quadrants in the equally cleaving polyclad embryo. Boyer *et al*. [73] identified the injected cells in *Hoploplana inquilina* retrospectively by comparing their contributions to the formation of larval ectoderm (and other cell fates, such as mesoderm formation), with those of other spiralian cell lineages. For example, as the D quadrant formed the dorsal axis in annelids and molluscs, the blastomere in polyclads that gives rise to dorsal ectoderm was identified as the D quadrant. However, this assumes that there is no variation among spiralian taxa, and as recent lineage studies have highlighted, there is a growing list of cell fate changes between taxa, even among the derivation of the mesoderm, which was once thought to be highly conserved [48,72]. In light of this variation, homologizing blastomeres - and homologizing structures based on blastomere origins - between the equally cleaving polyclads and other spiralian taxa may not be the most appropriate method for reconstructing the evolution of similar characters.

Marine life histories show tremendous variation with, for example, congeners that have long-lived feeding larvae and direct development [74,75]. This variation may be strongly influenced by phylogeny, or it may be a result of a species biogeographic distribution and environmental conditions [75,76]. In order to investigate the evolutionary and biogeographic dynamics of mode of development in polyclads, data are needed on the life history strategies of many more species at different latitudes and regions, and these life history data need to be considered within the framework of a robust molecular phylogeny. As all cotylean species investigated to date develop via an eight-lobed larva, it is probable that this is the ancestral condition for this suborder. However, it is too soon to determine whether similarities in larval structures among (and between) Götte’s and Müller’s larvae, are due to common ancestry or convergence.

A phylogeny of the polyclads, incorporating early branching taxa of both suborders (identifiable from existing hypotheses on polyclad inter-relationships [5,66,77]), would reveal the macroevolutionary distribution of developmental modes across families, and would shed light on the ancestral condition for the order. Species-level phylogenies of polyclad clades that show diverse modes of development (for example, the acotylean families: Notoplanidae, Stylochidae, Leptoplanidae) will allow us to investigate the rate and frequency of changes in mode of development. These phylogenies will also allow us to identify sister taxa with different developmental modes, which may be used for comparative studies of the developmental processes involved in the gain and/or loss of larval characters. Finally, understanding the intra-specific variation of larval phenotypes on which contemporary selective pressures and developmental, functional and physiological constraints act, will allow us to gain a better picture of the microevolutionary processes that generate macroevolutionary patterns in mode of development.

## Conclusions

There is still much that we do not know about polyclad larval diversity, development and evolution. With the mode of development described for less than 8% of known species, there are undoubtedly more life history strategies and larval morphologies that remain to be discovered. With a greater understanding of the diversity, ecology, evolution and development of polyclad larvae, we will gain insight into the functional significance of variation in larval characters, such as the number of larval lobes and the ciliary band configurations. I have stressed the need for a phylogeny of the polyclads as a framework to understand the distribution of larval features across clades, and to direct research into the morphological and developmental differences between direct and indirect-developing species. In order to examine the turnover of morphological features over ontogeny, we also need to devote time to developing larval culturing techniques, and we must take full advantage of intermediate developing species as models for studies in metamorphosis.

Polyclads offer an excellent system with which to explore the anatomical convergence of larval structures among flatworms and spiralians. Polyclads are the only flatworms from which experimentally accessible embryos can be readily obtained and reared outside of their protective egg capsule (due to techniques pioneered by Rooney and Boyer [78]), and may therefore provide valuable insight into the evolution of flatworm and spiralian development. Of the few polyclad species that can be collected in large numbers, the cotylean *M. crozieri* is emerging as a tractable system to study polyclad embryology and larval development [52,53]. The relative ease of collection in the field and extraction of naked embryos, its comparatively fast development and large egg size make *M. crozieri* a promising polyclad model species for evolutionary developmental studies. Transcriptomic and/or genomic resources currently available for a number of polyclad and other spiralian taxa are facilitating comparative studies of the molecular mechanisms underlying the development of larval characters. When considered in a phylogenetic framework, these developmental data will shed light on the nature of the evolutionary relationship between spiralian larval characters and their underlying developmental mechanisms.

## Competing interests

The author declares that she has no competing interests.
